# A Genetic Linkage Map of Sole (*Solea solea*): A Tool for Evolutionary and Comparative Analyses of Exploited (Flat)Fishes

**DOI:** 10.1371/journal.pone.0115040

**Published:** 2014-12-26

**Authors:** Eveline Diopere, Gregory E. Maes, Hans Komen, Filip A. M. Volckaert, Martien A. M. Groenen

**Affiliations:** 1 Laboratory of Biodiversity and Evolutionary Genomics, University of Leuven, Ch. Deberiotstraat 32, B-3000 Leuven, Belgium; 2 Animal Breeding and Genomics Centre, Wageningen University, Marijkeweg 40, NL-6700 AH Wageningen, the Netherlands; 3 Centre for Sustainable Tropical Fisheries and Aquaculture, James Cook University, 4811 QLD Townsville, Australia; Nanjing Forestry University, China

## Abstract

Linkage maps based on markers derived from genes are essential evolutionary tools for commercial marine fish to help identify genomic regions associated with complex traits and subject to selective forces at play during exploitation or selective breeding. Additionally, they allow the use of genomic information from other related species for which more detailed information is available. Sole (*solea solea* L.) is a commercially important flatfish species in the North Sea, subject to overexploitation and showing evidence of fisheries-induced evolutionary changes in growth- and maturation-related traits. Sole would definitely benefit from a linkage map to better understand how evolution has shaped its genome structure. This study presents a linkage map of sole based on 423 single nucleotide polymorphisms derived from expressed sequence tags and 8 neutral microsatellite markers. The total map length is 1233.8 cM and consists of 38 linkage groups with a size varying between 0 to 92.1 cM. Being derived from expressed sequence tags allowed us to align the map with the genome of four model fish species, namely medaka (*Oryzias latipes*), Nile tilapia (*Oreochromis niloticus*), three-spined stickleback (*Gasterosteus aculeatus*) and green spotted pufferfish (*Tetraodon nigroviridis*). This comparison revealed multiple conserved syntenic regions with all four species, and suggested that the linkage groups represent 21 putative sole chromosomes. The map was also compared to the linkage map of turbot (*Scophthalmus maximus*), another commercially important flatfish species and closely related to sole. For all putative sole chromosomes (except one) a turbot homolog was detected, confirming the even higher degree of synteny between these two flatfish species.

## Introduction

Preserving the evolutionary potential of exploited marine fish species is essential to secure viable populations across a species’ full geographical and environmental range [1]. A better understanding of the strength of local and fisheries-induced adaptation hence provides important insights into the resilience and evolutionary response to environmental change and harvesting of stocks [2]. This is crucial to move towards evolutionary enlightened sustainable fisheries management [3]. In the last decade, marine fisheries have strongly declined or even collapsed [4], [5] and a growing number of fish population studies have reported significant changes in life history traits that have been associated with fisheries-induced selection [6], [7], [8]. Examples include shifts towards earlier maturation at a smaller size, increased reproductive investment and changes in growth rate, all of which are consistent with the size-selective nature of fishing. These changes, in synergy with climate change, may have drastic negative effects on fish populations and their sustainable fisheries [2]. One of the most challenging problems in studying local adaptation and fisheries-induced evolution, however, is disentangling the environmental and genetic causes behind changes in life-history traits [7], [9], [10]. Many of the adaptive traits that respond to evolution are complex and quantitative by nature. Hence, genomic tools became pivotal to reveal footprints of selection, as changes can now be studied directly at the molecular level.

Genetic linkage maps represent one of such essential evolutionary genomics tools for commercial marine fish, to help identify genomic regions associated with complex traits subject to selective forces. Additionally they are crucial during selective breeding initiatives, aiming at relieving fishery pressure on overexploited stocks, while increasing the cost-efficiency of farmed fish production. They also provide the necessary resources for genomic comparison with other fish species to understand their genome evolution and organization [11], [12] and facilitate anchoring of scaffolds to chromosomes in whole genome sequencing and assembly [13].

Sole (*Solea solea* L.) is a commercially important marine flatfish (Pleuronectiformes) of the family Soleidae mainly living in the Northeast Atlantic Ocean, but also in the whole Mediterranean Sea and in the Southwestern Black Sea [14]. The spawning stock biomass of the North Sea has been fluctuating around the precautionary point of 35,000 tons depending on the strength of the year classes. Periods of revival however were short and overall there has been a downward trend, putting the sole stock at risk of reduced reproductive capacity [15]. Additionally, strong evidence exists for fisheries-induced evolutionary change in the onset of sexual maturity over the last 60 years [16].

Despite the commercial importance of sole, no genomic tools are available to date [17], [18]. Currently, genetic linkage maps are available for only five other flatfish species: turbot (*Scophthalmus maximus*) [19], [20], brill (*Scophthalmus rhombus*) [21], Atlantic halibut (*Hippoglossus hippoglossus*) [22], half-smooth tongue sole (*Cynoglossus semilaevis*) [23] and olive flounder (*Paralichthys olivaceus*) [24], [25]. One of the major obstacles to build a linkage map for sole has been the lack of informative genetic markers. However, cutting-edge next-generation DNA sequencing technologies allow for a rapid and cost-efficient development of genetic markers across the genome of highly exploited species without any existing genomic information [26].

Here, we used a novel set of single nucleotide polymorphisms (SNPs) that were developed from expressed sequence tags (ESTs) to construct the first linkage map of sole. Additionally, we used this map for comparative mapping and establishing the syntenic relationships with four fully sequenced model species, namely medaka (*Oryzias latipes*), Nile tilapia (*Oreochromis niloticus*), three-spined stickleback (*Gasterosteus aculeatus*) and green spotted pufferfish (*Tetraodon nigroviridis*). These comparisons were ultimately used as a stepping stone to compare the sole linkage map with that of turbot (*Scophthalmus maximus*), which is more closely related to sole than the model fish species. The future applications of this novel molecular tool were further discussed into the context of evolutionary based fisheries management of flatfish species.

## Materials and Methods

### Mapping families

Two full-sib families with 46 and 35 offspring respectively were used for the linkage analysis. The sampling of the parents, broodstock management, offspring collection and DNA extractions were done by Blonk [27] and were part of a large breeding program, initiated by Solea B.V. (The Netherlands) and the Animal Breeding and Genomics Centre (Wageningen University), aiming at increased productivity of farmed sole. All procedures were in accordance with the Dutch law. The parents originated from the Southern North Sea (52°N and 2.5°E) and were collected between 2003 and 2005. Details on the management of the broodstock including these parents (called ‘B’) and their offspring can be found in [28]. Of each parent a blood sample (0.1 ml) was taken without killing them (as they were part of the sole breeding program). DNA was extracted from this sample using a Puregene DNA purification kit for non-mammalian whole blood samples (Gentra Systems). DNA extraction of the offspring was performed on 3 to 4 days old larvae using nucleospin tissue columns following the manufacturer’s guideline (96 procedure, Machery-Nagel).

### Genetic markers and genotyping

Using the Roche FLX Titanium technology, in total 348,042 cDNA sequences were generated from a multiplexed sole muscle library based on seven sole individuals sampled across the East Atlantic Ocean and the Mediterranean Sea (unpublished data). A total of 11,021 contigs could be assembled and over 3,000 SNPs detected *in silico*. Among those, 1,536 SNPs were selected for validation using the Illumina GoldenGate™ high-throughput genotyping assay. Some of these markers have already been applied successfully in a traceability context [29]. The inheritance and the informativeness of the markers were visually checked with the GenomeStudio™ genotyping module of Illumina. Of the 1,536 genotyped SNPs, 749 were not informative within the two families. For 21 SNPs inconsistencies with mendelian inheritance were observed and for 297 SNPs the genotyping assay failed. An overview of the remaining 469 SNPs that were used to perform linkage analysis, can be found in S1 Table. In addition both families were genotyped at the following 10 microsatellite markers with the method described in Blonk et al. [28]: *AF173849*, *AF173852*, *AF173854*, *AF173855* [30], *AY950587*, *AY950588*, *AY950589*, *AY950591*, *AY950592*, *AY950593* [31]. In total 479 markers (469 SNPs and 10 microsatellites) were included in the linkage analysis. A high number of missing data often indicates either poor DNA quality or markers that are difficult to call, which may lead to difficulties in linkage map construction. Therefore, individuals or markers with more than 30% missing data were excluded from the dataset, being two individuals and none of the markers. The final dataset consisted of two families with respectively 46 and 33 offspring (with no more than 3% missing values per individual) and 479 markers (of which only 13 SNPs had more than 3% missing data).

### Linkage map construction and genome coverage

SNPs located within the same contig were combined into haplotypes using the program Pedphase v3.0 [32]. The map was built with the program Cri-map v2.4 [33]. Initial grouping of the markers was carried out using the *twopoint* and *autogroup* option of Cri-map. The *twopoint* analysis was performed for all pairs of markers with a threshold of LOD = 3. *Autogroup* was then used to identify sets of markers which were likely located in the same linkage group (LOD ≥8). Some small linkage groups were pooled based on additional information: (1) an *autogroup* analysis with a lower threshold (LOD of four instead of eight) and (2) if linkage was found between the majority of the markers after performing an additional *twopoint* analysis with a threshold of LOD = 0.5. Secondly, the position of the SNPs and microsatellite markers within the groups was determined with the *build* option of Crimap in an iterative process, starting with a LOD score of 3 and through subsequent stepwise lowering of the LOD score. Finally, marker order was calibrated using the *flips* option with a window size of five markers. The map was drawn in the program Mapchart v2.2 [34]. Two methods were used to estimate the genome length (G_e_) of sole. According to Fishman et al. [35] corrections should be made for chromosome ends by adding 2*D_av_ to the length of each linkage group, where D_av_ is the average inter-marker distance of the linkage map. Chakravarti et al. [36] suggested to multiply each linkage group by (m+1)/(m-1), where m is the number of loci in each linkage group (4th method in Chakravarti et al. [36]). The average length calculated by both methods was used as an estimate for the genome length (resp. G_e1_ and G_e2_).

### Comparative mapping and syntenic relationships

As all SNPs in the sole map were discovered from ESTs, the assembled contigs were used as queries to perform a local blast against the genome of four fully sequenced fish species: medaka (*Oryzias latipes*, order Beloniformes), Nile tilapia (*Oreochromis niloticus*, order Cichliformes), three-spined stickleback (*Gasterosteus aculeatus*, order Perciformes) and green spotted pufferfish (*Tetraodon nigroviridis*, order Tetraodontiformes). Of all available model species, these four are most closely related to the Pleuronectiformes, each representing a different order within the Percomorphaceae [37]. Genomic information was downloaded from the genome browser Ucsc (http://genome.ucsc.edu/). A Blastn analysis was performed with NCBI-Blast under default settings with exception of the E-value (<10^−10^). To establish syntenic relationships with turbot (*Scophthalmus maximus*) a direct comparison by blast analysis was not possible, as this species is not fully sequenced yet. Therefore, the stepping stone approach as described by Sarropoulou et al. [38] was used. The four model species were used as a bridge which allowed passing from the sole linkage groups to those of turbot.

## Results and Discussion

### Linkage map construction and genome coverage

**Figure 1:**
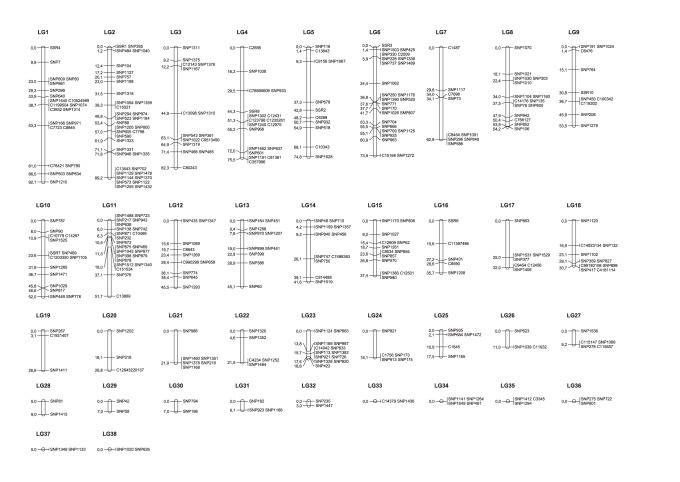
Sex-averaged linkage map of sole. Map distances are calculated using the Kosambi mapping function and shown in centimorgans. Combined SNPs are indicated with a ‘C’ at the beginning of their name.

The 469 informative SNPs were distributed over 291 single-marker contigs and 73 contigs with multiple SNPs. These markers located on the same contig were combined into haplotypes, in order to compensate for the low number of informative meioses due to the low number of individuals in the mapping families and to provide a more accurate estimate of the recombination frequency between the markers [39]. This was done for all contigs with multiple SNPs, except for four of them because of evidence for recombination within that contig (S1 Table). Of the 469 SNPs and 10 microsatellite markers, 423 and 8 (∼90%) respectively were incorporated into the linkage map (Fig. 1, S1 Table). The total length of the map was estimated at 1233.8 cM and consists of 38 linkage groups (LG1 to LG38) with a size varying between 0 to 92.1 cM (Table 1, S1 Table). The number of markers per group ranges from two to 39. The average inter-marker distance (D_av_) is 8.1 cM and the maximal interval is 32.7 cM. The 48 unmapped markers could not be assigned a position due to an insufficient number of informative meioses. For the same reason, the order of closely linked markers might be inaccurate or it may be impossible to separate markers.

**Table 1:** Number of markers, the corresponding number of distinct contigs and map length for each linkage group of sole.

LG	No. Markers	No. contigs	Length (cM)
LG1	30	21	92.1
LG2	39	35	89.2
LG3	21	15	82.3
LG4	32	17	75.5
LG5	15	10	74.8
LG6	28	25	73.9
LG7	12	9	62.8
LG8	17	15	54.2
LG9	15	9	53.5
LG10	18	14	52
LG11	27	17	51.7
LG12	14	10	45.5
LG13	10	10	45.1
LG14	14	11	41.6
LG15	16	13	37.4
LG16	8	4	35.7
LG17	9	6	32
LG18	16	10	30.7
LG19	4	3	26.9
LG20	7	3	26.8
LG21	6	6	21.9
LG22	6	5	21.5
LG23	14	13	18.8
LG24	6	5	18.1
LG25	6	5	17.5
LG26	4	3	11
LG27	8	5	9.2
LG28	2	2	9
LG29	2	2	7
LG30	2	2	7
LG31	3	3	6.1
LG32	2	2	3
LG33	3	2	0
LG34	4	4	0
LG35	4	3	0
LG36	3	3	0
LG37	2	2	0
LG38	2	2	0

The estimated genome length according to Fishman et al. [35] (G_e1_) is 1849.4 cM. However, to obtain a more accurate estimate, two further corrections were made: (1) The distance between two markers in a linkage map should not exceed 30 cM [40]. As the haploid chromosome number of sole is 21 [41], there are 17 linkage groups in excess. For these 17, no correction was made for chromosome ends, but instead 17*30 cM was added to the map length of 1233.8 cM. (2) For the number of acrocentric chromosomes, 13 in the case of sole [41], only D_av_ instead of 2D_av_ was added (105.3 cM instead of 210.6 cM). For the other 8 chromosomes 2D_av_ was added (129.6 cM). In total, the estimated genome length (G_e1_) after both corrections is 1978.7 cM. The estimated genome length (G_e2_) according to the fourth method of Chakravarti et al. [36] is 1490.2 cM. The average of both values (G_e1_ and G_e2_) is 1734.45 cM, corresponding to approximately 70% genome coverage. Half of the linkage groups are larger than 30 cM, a size similar to those found in maps of other (flat)fish species [20], [22], [24], [42]. Twelve linkage groups are smaller than 10 cM, therefore covering only part of the chromosomes represented by these linkage groups. Several of these linkage groups most likely represent different parts of the same chromosomes.

### Comparative mapping with model fish species

The 423 mapped SNPs originated from 326 different contigs (S1 Table). The sequences for these contigs were aligned against the genome of stickleback, pufferfish, medaka and tilapia using Blastn. One-hundred and eighty contigs showed a significant sequence homology above the threshold (E-value <10^−10^) in at least one of the four fish species examined: 152 in stickleback, 126 in tilapia, 105 in medaka and 97 in pufferfish. Fifty-eight contigs showed a significant sequence homology in all four model species. A detailed overview of the Blastn results can be found in S2–S5 Tables. The highest levels of sequence similarity were found with the stickleback genome (∼47%), followed by the tilapia genome (∼39%) and the lowest levels with the medaka (∼31%) and pufferfish (∼30%) genome. However, these results are not in concordance with the phylogenetic position of the Pleuronectiformes among other Percomorphaceae, [37], [43]. Flatfish belong to the Carangimorphariae, which are most closely related to the Ovalentariae, which include medaka and tilapia, and more distantly related to Percomorpharia, which include stickleback and pufferfish. Similar results were observed for turbot [20]. Despite the closer relationship between turbot and medaka, more sequence similarity was observed with stickleback (∼50%) than with medaka and pufferfish (∼40%). Bouza et al. [20] suggested that this reflected phylogenetic discordances between the use of mitochondrial and nuclear genes, as the proposed phylogeny was based on the mitogenome [44], [45]. However, recent phylogenetic studies using a combination of both marker types do not support such discordance [37], [43], [46]. Another possible explanation for this apparent discrepancy is provided by the different evolutionary rates among closely related species. Medaka and pufferfish (and teleosts in general) evolved faster than other vertebrate species [45], [47], [48], [49], hence they might also have evolved faster than sole, stickleback and tilapia. Support for this is presented by Betancur et al. [43], which show that the branch lengths in their evolutionary tree are longer for medaka and pufferfish in comparison to the other three species. S6 Table lists the number and length of the conserved syntenic regions (i.e. markers on the same linkage group that are also on the same chromosome, regardless the order) with each of the four model species. Most syntenic sequences were found with stickleback (23 syntenic regions, containing on average 5.3 sequences), followed by tilapia, medaka and pufferfish (resp. 23, 19 and 18 regions, containing 4.3, 4.3 and 3.6 sequences). These numbers are in concordance with the observed levels of sequences similarity for each species (see above). The total length of the syntenic regions was smaller for stickleback (200.1 Mb) than for tilapia (257.8 Mb) and medaka (224.7 Mb), but larger when compared to pufferfish (89.2 Mb). This suggests that the stickleback genome is more compact in comparison to tilapia and medaka, but larger than pufferfish, which is consistent with their genome size. Tilapia has the largest genome at about 820 Mb, followed by medaka (∼700 Mb), stickleback (∼460 Mb) and pufferfish (∼350 Mb).

### Syntenic relationships between sole and model fish species

**Figure 2:**
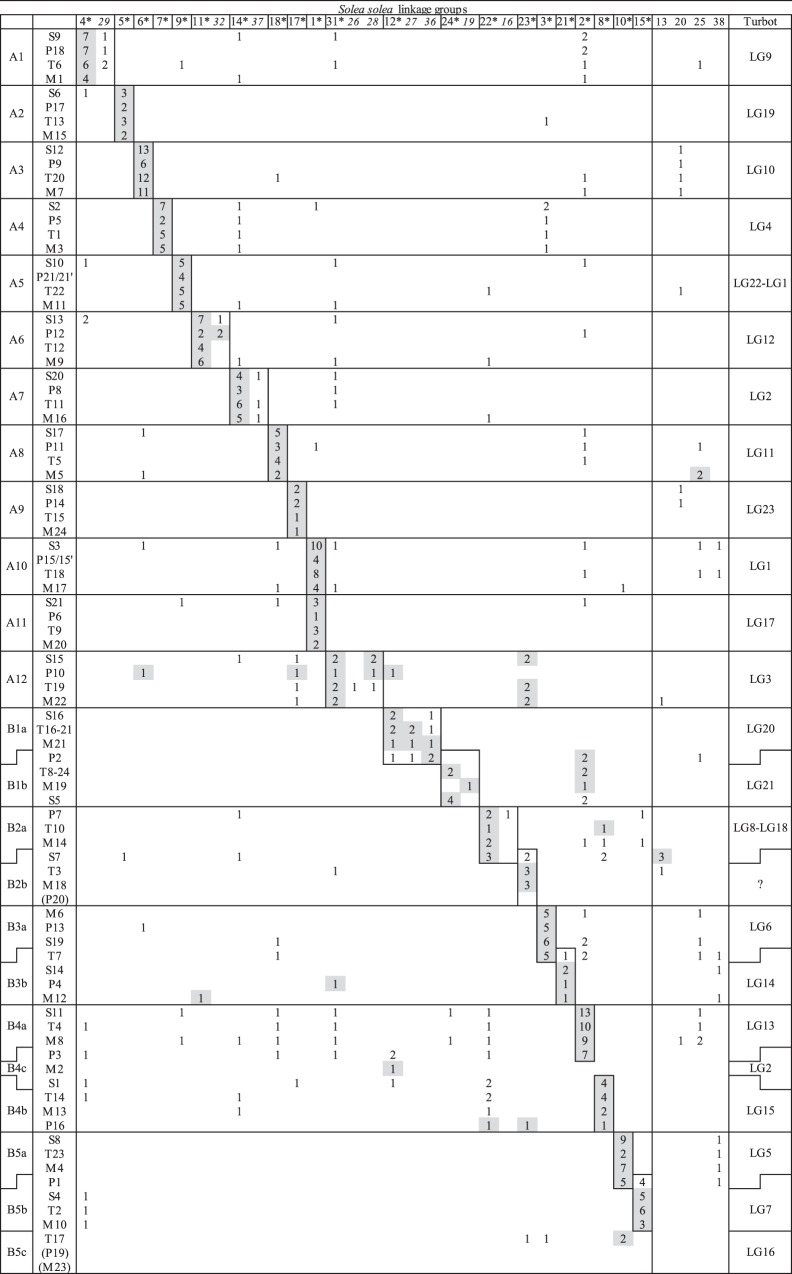
Syntenic relationships between sole and five other (flat)fish. The chromosomes of four model fish species, namely stickleback (S), tilapia (T), pufferfish (P) and medaka (M), were grouped in A- and B-groups according to their syntenic relationships as described in Sarropoulou et al. (2008), Kai et al. (2011) and Guyon et al. (2012) (left column). The numbers in the grid indicate the number of contigs where sequence homology was found between the sole linkage groups and the chromosomes of the four model species. For each chromosome the sole linkage group with the largest number of homologous sequences is highlighted in grey. Marked with *: the 21 linkage groups that are suggested as chromosome counterpart for sole (or at least part of it). In italics: linkage groups likely to be on the same chromosome as the linkage group marked with * to the left of it. For all 21 putative sole chromosomes (except for LG23) a homologous turbot linkage group is suggested (right column).

The distribution of the sequence homology between the model species chromosomes and the sole linkage groups is shown in Fig. 2. The chromosomes of the four model species are organized in A-groups (with one-to-one relationships) and B-groups (with inter-chromosomal rearrangements) according to their syntenic relationships as described in Sarropoulou et al. [38], Kai et al. [13] and Guyon et al. [50] (see left column). The middle section shows the number of contigs where sequence homology was found between the sole linkage groups and the chromosomes of the four model species. For each chromosome the sole linkage group with the largest number of homologous sequences is highlighted in grey. For the chromosomes belonging to the same A-group or B-subgroup this was mostly the same linkage group, pointing to the chromosome counterpart (or at least part of it) in sole. In total, twenty-one linkage groups were suggested as chromosome counterpart (marked with *). The remaining 17 linkage groups were in surplus as there are only 21 sole chromosomes [41]. Even with a limited number of blast hits (because of their smaller size), nine of these 17 (in italics) were suggested to be on the same chromosome as some of the other 21 linkage groups, as only hits were found with a single A- or B-subgroup.

The comparative analysis implies a high degree of conserved synteny, in addition to several chromosomal rearrangements. Twelve of the 21 putative sole chromosomes predominantly showed a one-to-one syntenic relationship with the model species, namely LG4_/29_-A1, LG5-A2, LG6-A3, LG7-A4, LG9-A5, LG11_/32_-A6, LG14_/37_-A7, LG18-A8, LG17-A9, LG12_/27/36_-B1a, LG2-B4a and LG8-B4b. Six other putative sole chromosomes also showed a one-to-one syntenic relationship, with the exception of one model species: LG22/16 and LG23, both showed homology with chromosome seven of stickleback (S7), indicating that the fusion event leading to S7 in the stickleback ancestor [38], did probably not occur in the sole ancestor. A similar observation was found between LG3, LG21 and chromosome seven of tilapia (T7), and between LG10, LG15 and chromosome one of pufferfish (P1), indicating that the fusion events leading to T7 and P1 [13], [50] did not occur in sole. Beside these three inter-chromosomal rearrangements between sole and one of the four model species, interestingly, one major inter-chromosomal rearrangement between sole and all four model species was observed, namely LG1, which corresponded to two A-groups (A10 and A11), suggesting a fusion in the lineage leading to sole. Finally, the remaining two putative sole chromosomes, LG31_/26/28_ and LG24_/19_, showed conserved synteny with the chromosomes belonging to the synteny groups, A12 and B1b, respectively. However, for A12 additional hits were found with LG23 and for B1b with LG2. The homology of LG23, LG2 and several other linkage groups with multiple chromosomes can be attributed to the presence of contigs that were annotated with multigene families (*e.g. myosine heavy chain*, *alpha-actinin-3-like*, *ryanodine receptor*, *major histocompatibility complex* and *tubulin* genes). Paralogous sequences from such families are dispersed among chromosomes and therefore interfere with the true syntenic relationships. For all 21 putative sole chromosomes one synteny group (or two in the case of LG1) was suggested, leaving B4c and B5c. In medaka (n = 24) they represent two additional chromosomes, M2 and M23 [47]. However, M2 merged together with M8 in pufferfish (P3) and with M13 in stickleback (S1), and M23 with M10 in stickleback (S4) [13], [38]. Also in tilapia several arguments suggested that M2 merged with M4 into T23 [50]. Unfortunately, for sole no blast hits were found with M2 nor M23 (except one between M2 and LG12).

### Syntenic relationships between sole and turbot

Combining linkage groups in sole chromosomes based on synteny with distantly related model species (*i.e.* all belonging to different orders within the Percomorphaceae), has to be done with cautious. Therefore, the syntenic relationships were also studied with another flatfish species, and thus much closer related. The sole linkage map was built exclusively with EST-based SNP markers (with the exception of eight anonymous microsatellite markers). Given their higher evolutionary conserved status than anonymous markers [51], [52], they provide a suitable framework for comparative mapping with fully sequenced species, as illustrated in the present study. However, as all markers are sole-specific and not present in the turbot or any other flatfish map, a direct comparison, similar to turbot and brill (*Scophthalmus rhombus*) [21], is impossible. The syntenic relationships between sole and turbot were established indirectly through evolutionary conservation with the model species [38], [53]. Bouza et al. [20] studied the syntenic relationships between turbot and stickleback, medaka and pufferfish. We used these three stepping stone species to anchor the sole linkage groups to turbot. For all 21 putative sole chromosomes (except for LG23) a homologous turbot linkage group was found (see right column in Fig. 2), suggesting a high degree of conserved synteny between these two flatfish. Additionally, these results support the approach of combining linkage groups based on synteny with model species and putting forward 21 putative sole chromosomes. However, also some differences were discussed below. For LG22_/16_ two turbot linkage groups (LG8-LG18) were observed, but it was suggested that these two probably are located on the same turbot chromosome. This was justified as LG8 and LG18 were syntenic to a single chromosome in all model species studied by Bouza et al. [20]. Moreover, the turbot map contains 24 linkage groups, so there are still two in surplus, as there are only 22 turbot chromosomes. Next, Bouza et al. [20] suggested a translocation between LG1 and LG22 in the lineage leading to turbot. This was not observed between the corresponding linkage groups of sole (resp. LG1 and LG9). For LG23 of sole (homologous with the chromosomes of synteny group B2b), no turbot homolog was found, whereas for LG16 of turbot (homologous with the chromosomes of synteny group B5c), no sole counterpart was suggested. These findings could be a consequence of different chromosomal rearrangements in the lineages leading to both species, however, more evidence is needed to confirm this. Finally, the fusion that was suggested leading to LG1 of sole, was clearly not observed for turbot. Interestingly, this is consistent with the karyotype of both species and might explain why sole has one chromosome less than turbot [54].

### Future applications

The fisheries-induced evolutionary change observed in North Sea sole over the last 60 years [16] might significantly impact the productivity of the stock, and eventually lead to stock collapse. From a conservation point of view, evolutionary insights are of paramount importance to avoid irreversible loss of adaptive genetic diversity. This linkage map based on gene-linked markers allowed for a first exploration of the sole genome. By studying the syntenic relationships with other (flat)fish, these genetic markers were positioned and 21 sole chromosomes were suggested. This knowledge is highly relevant for evolutionary studies and can serve as a roadmap to help identify and locate genes involved in local adaptive differentiation and fisheries-induced selection. Using genome scans and if selection is strong enough, genetic markers in the proximity of these genes will display reduced variation and higher degrees of differentiation (e.g. “selective sweeps”) [10], [55]. Identifying such genomic regions is impossible without knowledge on the location of the genetic markers. From a commercial point of view, a linkage map is an essential tool for selective breeding initiatives, aiming at relieving fishery pressure, while increasing the cost-efficiency of farmed fish production. The long generation time, slow growth and the occurrence of infections such as Black Patch Necrosis, have slowed down the large-scale aquaculture of sole [56]. However, research on the culture of various sole species has gained momentum [18], [57], leading to novel breeding strategies, optimised sustainable feed and improved disease resistance. The promising results from recent experimental selective breeding initiatives of sole [58], [59] further stimulate the development of genomic tools in the field of aquaculture. Maximizing growth is a focal goal in aquaculture. For turbot, a more successful flatfish in aquaculture than sole, knowledge on the locations of several growth-related traits is already available. Given the comparison between turbot and sole, that was provided by the present study, the available genomic information of turbot might point in the direction of growth-related genomic regions in sole, similar as was recently illustrated by Hermida et al. [21] between the two flatfish, turbot and brill.

## Conclusion

This study constitutes the first mapping effort of the sole genome using gene-linked markers. This linkage map provides a good genome coverage with a homogenous distribution of the markers. Although genomic resource are limited for sole, this map allowed us to explore genomic architecture through comparative mapping with other fish species. The EST-linked markers offered a useful framework for a comparison with four model species. A remarkable degree of conserved synteny was observed, enabling to reconstruct 21 putative sole chromosomes with a high degree of confidence. Several cases of inter-chromosomal rearrangements were suggested as well, including a fusion in the lineage leading to sole. The stepping stone approach, used to compare the sole and turbot genome, confirmed the even higher degree of conserved synteny between the more related flatfish species. For all putative sole chromosomes (except one) it was possible to detect a turbot homolog. The fusion in sole was not observed in turbot, which is consistent with their karyotypes. This first sole map and comparative analysis represents a good starting point to identify functional genomic regions and associated candidate genes of evolutionary and commercial interest in sole, but also in other Pleuronectiformes and teleosts.

## Supporting Information

S1 Table Details on the genetic markers: marker name, linkage group, position (cM), corresponding contig name and accession number.
(XLSX)

S2 Table Blast results of sole sequences against the stickleback genome.
(XLSX)

S3 Table Blast results of sole sequences against the pufferfish genome.
(XLSX)

S4 Table Blast results of sole sequences against the medaka genome.
(XLSX)

S5 Table Blast results of sole sequences against the tilapia genome.
(XLSX)

S6 Table Summary of sole linkage groups and homologous stickleback, pufferfish, medaka and tilapia chromosomes that share at least two markers (i.e. conserved syntenic regions).
(XLSX)
